# MeHA: A Computational Framework in Revealing the Genetic Basis of Animal Mental Health Traits Under an Intensive Farming System—A Case Study in Pigs

**DOI:** 10.3390/biology13100843

**Published:** 2024-10-21

**Authors:** Jinyun Jiang, Lingyao Xu, Yizheng Zhuang, Xingyu Wei, Zhenyang Zhang, Wei Zhao, Qingyu Wang, Xiaowei Ye, Jiamin Gu, Caiyun Cao, Jiabao Sun, Kan He, Zhe Zhang, Qishan Wang, Yuchun Pan, Zhen Wang

**Affiliations:** 1 College of Animal Sciences, Zhejiang University, Hangzhou 310058, China; jjinyun@zju.edu.cn (J.J.); 22217005@zju.edu.cn (L.X.); 22317058@zju.edu.cn (Y.Z.); 22117096@zju.edu.cn (X.W.); zhangzhenyang@zju.edu.cn (Z.Z.); wangqingyu@zju.edu.cn (Q.W.); ye_xw@zju.edu.cn (X.Y.); 12117016@zju.edu.cn (J.G.); ccyun@zju.edu.cn (C.C.); sunjiabao@zju.edu.cn (J.S.); zhe_zhang@zju.edu.cn (Z.Z.); wangqishan@zju.edu.cn (Q.W.); panyc@zju.edu.cn (Y.P.); 2 SciGene Biotechnology Co., Ltd., Hefei 230031, China; wayne-zhao@foxmail.com; 3 Center for Stem Cell and Translational Medicine, School of Life Sciences, Anhui University, Hefei 230601, China; hekan_803@163.com; 4 Traditional Chinese Medicine Research Centre, School of Life Sciences, Anhui University, Hefei 230601, China; 5 Hainan Institute of Zhejiang University, Sanya 572025, China

**Keywords:** computational framework, mental health, animal welfare, brain

## Abstract

Intensively farmed animals such as pigs inevitably experience a certain degree of psychological stress, which leads to a reduction in production performance. Mental health traits are currently difficult to measure, resulting in a gap in understanding their genetic basis. To address this challenge, we propose a computational framework called mental health of animals (MeHA), capable of revealing genes related to animal mental health traits. Using MeHA, we identified 109 candidate genes associated with pig mental health and discovered their intricate connections with critical functions, such as memory, cognition, and neural development, which are essential components of mental health and cognitive performance. Importantly, our findings provide evidence of the potential impact of these genes on economically important traits, including meat quality and piglet survival. This research underscores the importance of genetic studies in enhancing our understanding of animal behavior and cognition, as well as promoting agricultural practices. By applying our approach to study the genetic basis of mental health in pigs as a case, we confirmed that our framework is an effective way to reveal genetic factors affecting animal mental health traits, which contributes to animal welfare and has potential implications for understanding human mental disorders.

## Simple Summary

In this study, we developed a computational pipeline named MeHA to identify candidate genes related to mental health in animals within intensive farming environments, even in the absence of phenotype data. By applying MeHA to pigs, we identified 109 candidate genes associated with memory, cognition, and neural development that also influence traits such as meat quality. Our findings underscore the significance of genetic research in elucidating animal behavior, enhancing farming practices, and illuminating mental well-being in both porcine species and humans. This work illustrates the critical connection between genetics, behavior, and productivity in animal husbandry.

## 1. Introduction

As the global population grows and economic development progresses, intensive farming systems have become the main approach to meet the demand for meat [1]. To meet the needs of scaling-up farming operations and increasing the product yield per unit area, as well as to ensure product quality control, humans have long engaged in artificial selection and domestication, transforming wild species into breeds suitable for intensive farming [2]. These intensive animal breeds have started to adapt to intensive farming environments, exhibiting characteristics such as rapid growth and the ability to thrive under high-density conditions that meet human requirements [3]. However, intensive farming also poses challenges to animal welfare. For example, traditional farming methods generally involve small-scale extensive or semi-intensive farms with a high level of personal involvement of the farmer, allowing animals greater freedom of movement. Animals in these systems have longer breeding periods, and fattening animals have a longer lifespan [4], which aligns more closely with animal welfare requirements. In contrast, intensive farming environments may introduce new stressors (such as overcrowding, weaning, or mixing), which can trigger stress responses in animals [5] and negatively impact their health and productivity. For example, studies have shown that high stocking densities can cause stress in pigs, leading to various diseases, including mental disorders and even death [6,7]. There is a lack of consensus regarding a universally accepted definition of ‘mental health’ [8,9,10]. Moreover, the term ‘mental health’ is frequently used interchangeably with ‘mental illness’ in contemporary discourse [11]. The concept of mental health can be understood in different ways: it can be defined as the absence of mental disease [12], or it can encompass a broader state of well-being that incorporates biological, psychological, and social factors influencing an individual’s mental state and ability to function effectively within their environment [13,14].

The choice of animals as research subjects in the study of mental health poses greater challenges than the use of humans as research subjects, primarily for two reasons. First, some aspects of mental health disorders, such as the experience of involuntary intrusive memories of a traumatic event, may not be easily studied in animals [15]. Second, it is difficult to assess phenotypic data in animal research [16], making it challenging to determine emotions such as fear, sadness, and anxiety in animals. Therefore, we have opted to study pigs, which have brain structures that are more similar to those of humans [17]. Our aim is to develop a method that allows us to analyze and assess changes in mental health without relying on phenotypic data. This research will serve as a reference for studying mental health changes in other animals during the transition from wild to intensively farmed animal breeds.

Wild boars are known for their robustness, strong adaptability [18], and other economic trait characteristics, such as a lower carcass weight and higher intramuscular fat content [19]. Over time, humans have selectively domesticated wild boars into local pigs to increase specific traits, such as the lean meat percentage and growth rate, leading to improvements in important economic traits, such as body length, weight, and lean meat percentage [20]. Moreover, humans have gradually shaped the genomes of wild boar populations to coexist with those of humans through the selective breeding of individuals with more docile temperaments and desired characteristics [21]. Duroc, Landrace, and Yorkshire pigs, as representative breeds of intensively farmed pigs, were developed through the hybridization of Near Eastern pigs—ancestors of Anatolian wild boars, domesticated 10,500 years ago—and European wild boars [22]. Since the first domestication events, pigs have been subjected to artificial directional selection [23]. Over more than a century of intensive selection, several traits have changed, such as those identified by Yu et al., which revealed positive selection in terms of growth and meat quality [24]. Given this background, we can postulate a plausible hypothesis: even in the absence of direct selection for mental health traits, there may have been a form of passive selection during the evolutionary transition from wild boars to modern intensively farmed breeds. This passive selection process could have inadvertently eliminated pigs exhibiting hypersensitivity traits that negatively impact production or the reproductive performance, thereby shaping the genetic basis of pig mental health traits. Research has shown that mental health traits can significantly influence economic traits through various mechanisms, including daily weight gain and a productive lifespan [25]. By delving into the genomic comparison between European wild boars and their intensively bred descendants, we can gain valuable insights into the genetic factors that influence mental health traits in pigs under intensive farming systems. These findings are crucial as they could provide feasible approaches for studying the genetic factors affecting mental health traits in other animals during their evolutionary process. Moreover, addressing mental health issues in animals could improve animal welfare and have profound implications for the economic viability and long-term sustainability of the livestock industry as a whole.

Therefore, in response to the prevailing trend of intensive farming environments, we have developed a computational framework called MeHA in this study to identify candidate genes for mental health in animals. We applied MeHA to pigs by leveraging the power of whole-genome sequencing (WGS) to delve into the genetic architecture of European wild boars and their intensively bred counterparts, with a specific focus on identifying genes that are associated with mental health traits in pigs. MeHA aims to shed light on the genetic underpinnings of animal mental health, which is a crucial yet often overlooked aspect of animal welfare in intensive farming systems. By utilizing this approach to identify candidate genes related to mental health, it becomes possible to enhance animal breeds, or prevent mental disorders and abnormal behaviors in animals at the genetic level. This contributes to improving productive traits and economic benefits. Our study represents a significant step forward in our quest to understand and improve the lives of animals in intensive farming environments, and we anticipate that the knowledge generated will have a profound and lasting impact on animal welfare and genetics.

## 2. Materials and Methods

**Figure 1:**
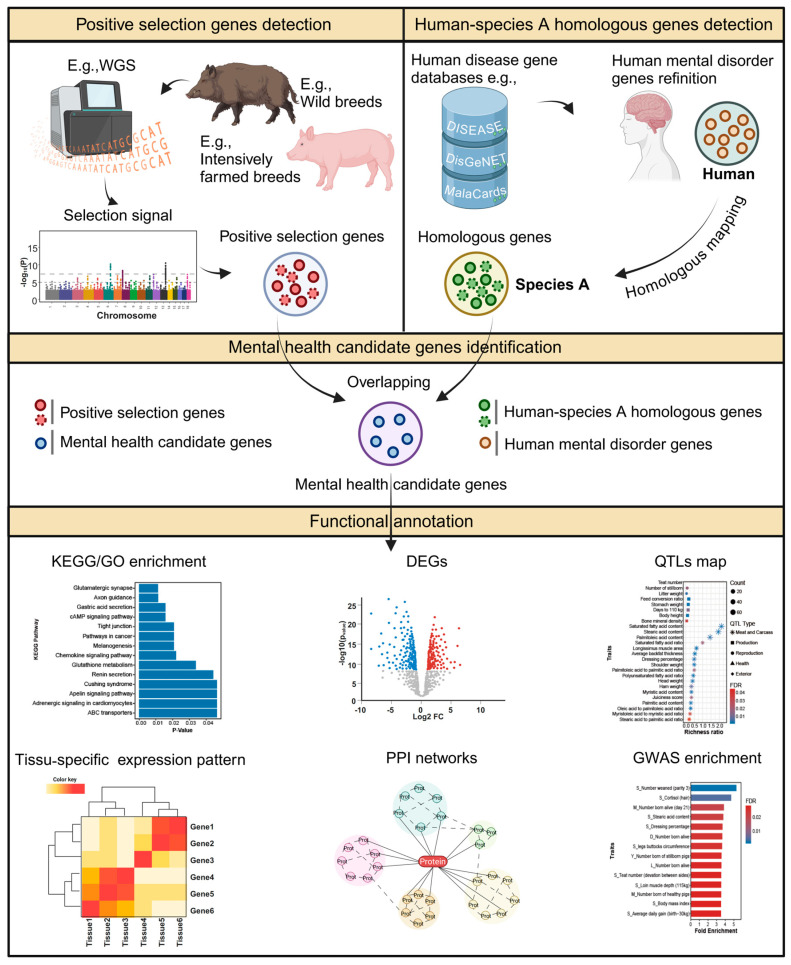
Framework of animal mental health candidate gene identification. Created with BioRender.com.

In this study, we developed MeHA (Figure 1) to identify mental health candidate genes in intensively farmed animals. In this framework, we can first use sequencing methods such as WGS to obtain genomic data, followed by a series of data filtering and processing steps. Combined with various selection signal analyses, we can identify positively selected genes. Since animals lack phenotype data related to mental disorders, while there is ample research on human mental illnesses, we mined common genes from multiple human disease gene databases, defining them as human mental disorder genes. Subsequently, through homologous conversion, we can obtain homologous genes for the desired species. By taking the overlapping portion of positively selected genes and homologous genes, we can obtain mental health candidate genes. Here, we applied MeHA to pigs by integrating pig genomic data and human mental disorder data to identify PMH candidate genes and systematically investigated their characteristics and functions.

### 2.1. Samples and Genotypes

We collected the genotypes of 170 pigs (Table S1), including three intensive pig breeds (Duroc, D, n = 49; Landrace, L, n = 47; Yorkshire, Y, n = 35), European wild boar (EWB, n = 21), and Asian wild boars (AWB, n = 18) from the PHARP v2 database [26], a pig genotype source containing approximately 23.1 million SNPs, and a total of 2048 individuals involved intensively farmed breeds and wild boars. To ensure the quality and independence of the genotype data, we further filtered the genetic variation data. Initially, we configured the parameters --maf 0.05 and --hwe 0.000001 to screen out loci with a gene frequency below 0.05 and a Hardy–Weinberg equilibrium test *p*-value under 0.000001. The adoption of these parameters serves to ensure that our analysis is founded on genetic variations with appropriate frequencies and equilibrium, thereby enhancing the reliability and precision of our research findings. Next, we used Plink v1.9 [27] software with the “independent-pairwise 20 2 0.3″ parameters to calculate the linkage disequilibrium (LD) between variants. This step helps to identify variants that are in high LD with each other, which can lead to redundancy in the dataset. Finally, a total of 823,055 SNPs were retained for subsequent analyses.

### 2.2. Population Genetic Structure Analysis

To assess the population’s genetic structure, we employed three approaches, including principal component analysis (PCA), phylogenetic tree construction, and admixture, to investigate the genetic relationships within individuals. First, we performed a PCA via the “--pca 10” parameter in Plink v1.9 software [27]. Second, we also used Plink v1.9 [27] to calculate the identical-by-state (IBS) matrix, which measures the genetic similarity between individuals. The genetic distance matrix was used to construct a neighbor-joining tree via the ape package [28] with the “bionj” parameter. Third, we employed Admixture (1.3.0) [29] software to estimate the population purity and the proportions of shared ancestry. The admixture population structure estimation was conducted for scenarios where the value of K ranged from 2–5. To validate the clustering results, we employed the GCTA (V1.94) software package [30] to compute kinship coefficients among individuals. Subsequently, we utilized R to visualize the genetic relationship matrix (G matrix).

### 2.3. Signal Detection

To detect the genomic region under selection, we employed Fst [31] and XP-EHH [32,33] approaches to identify potential selection signals. We set the EWB population as the common reference population and the intensive pig population as the observation population to calculate the Fst and XP-EHH values. These approaches allowed us to identify potential selection signals in the genomic regions and prioritize candidate genes that may be under positive selection in the intensive pig population compared with the EWB population.

#### 2.3.1. Fst Test

We used VCFtools software (0.1.16) [34] with the parameter “--weir-Fst-pop ” to calculate the population differentiation index (Fst) between the EWB and the intensive pig population. To minimize errors, we used a sliding window method with a window size of 50 kb and a step size of 10 kb. The genomic regions were then ranked on the basis of the mean Fst value, and the top 1% of the regions were identified as putative selection signals.

#### 2.3.2. XP-EHH Test

We used “selscan” (V1.2.0) software [35] to calculate the XP-EHH values and considered the genomic regions with the top 1% XP-EHH values as putative selection signals.

### 2.4. Putative Candidate Genes Under Selection

We considered genes within the 10 kb region upstream and downstream of the identified selection signatures as potentially selected candidate genes and utilized the VEP [36] with the pig reference genome Sscrofa11.1 [37] to annotate the selection signals.

### 2.5. PMH Candidate Gene Identification

Despite the challenges in identifying genes associated with mental health traits in pigs due to the limitation of phenotyping those traits, extensive research has been dedicated to identifying such genes in humans [38,39,40].

Recognizing the pig as a valuable model organism for human biology, we extensively engaged with human disease databases. Through a meticulous pig‒human homology analysis, we curated a preliminary set of reference genes pertinent to human mental disorders in our other study (unpublished) (Table S2), which encompassed a total of 1549 pig‒human homologous genes. Briefly, we systematically collected and curated gene‒disease annotation information from human disease databases, including DISEASE, DisGeNET, and MalaCards. The overlap between the candidate genes under selection and the pig–human homologous genes, which are known to be associated with mental disorders in humans, has been identified as the pig mental health (PMH) candidate genes.

### 2.6. Characterization of PMH Candidate Genes

#### 2.6.1. Functional Enrichment Analysis

We utilized the KOBAS [41] web server (accessed on 8 January 2024) (http://bioinfo.org/kobas) to identify the kyoto encyclopedia of genes and genomes (KEGG) pathways and gene ontology (GO) terms enriched with PMH candidate genes. *p* values were adjusted for multiple tests via the FDR [42,43] method, and the adjusted *p* value was set to 0.05 as the significance threshold.

Subsequently, we defined the 1337 genes eliminated during the screening process from positively selected genes to PMH candidate genes as eliminated genes. We then conducted a disease enrichment analysis on the PMH candidate genes and eliminated genes using the KOBAS website, including OMIM [44], KEGG Disease [45], and NHGRI GWAS Catalog databases [46]. By comparing the enrichment levels of various mental disorders in the results of both sets, we demonstrated the feasibility and accuracy of the MeHA method.

#### 2.6.2. Tissue-Specific Gene Expression Analysis

To study the tissue-specific expression of candidate genes related to pig mental health, we utilized the transcriptomic expression data of pig brain tissues from the Pig-GTEx database [47]. We employed the Euclidean distance as the distance metric and performed hierarchical clustering analysis via Ward’s method. This analysis clusters genes with different expression levels in tissues into different clusters. To visualize the gene expression patterns, we generated a heatmap via the “heatmap.2” function from the R “gplots” package. This analysis provides insights into the tissue-specific expression patterns of the candidate genes and their potential relevance to pig mental health.

#### 2.6.3. QTL Mapping Analysis

We utilized the Pig quantitative trait locus (QTL) Database [48] to annotate economically relevant traits associated with PMH candidate genes. To perform annotation and visualization analyses, we utilized the “GALLO” package and “ggplot” in R. This analysis helps us understand the potential genetic basis of economically important traits and their associations with PMH candidate genes.

#### 2.6.4. GWAS Signal Enrichment Analysis

To investigate the relationship between PMH candidate genes and production traits, providing a reference for future studies, we employed GWAS signal enrichment analysis via the PigBiobank database [49]. The *p* value was calculated using the formula [50]:

$$\begin{array}{c} P=1-F(x-1|N,M,N)=1-\sum_{i=0}^{x-1}\frac{\left(\begin{array}{l} n\\ i \end{array}\right)\left(\begin{array}{l} N-n\\ M-i \end{array}\right)}{\left(\begin{array}{l} N\\ M \end{array}\right)} \end{array}$$

*N* refers to the total number of genes in all background traits, *n* represents the genes identified in mental health traits, *M* denotes the genes identified in background traits, and *x* stands for the number of overlapping genes. This analysis allows us to assess whether the candidate genes identified in the context of pig mental health also show significant associations with other economic traits. We considered the FDR < 0.05 as the threshold for significantly correlated traits.

## 3. Results

### 3.1. Population Genetic Structure

**Figure 2:**
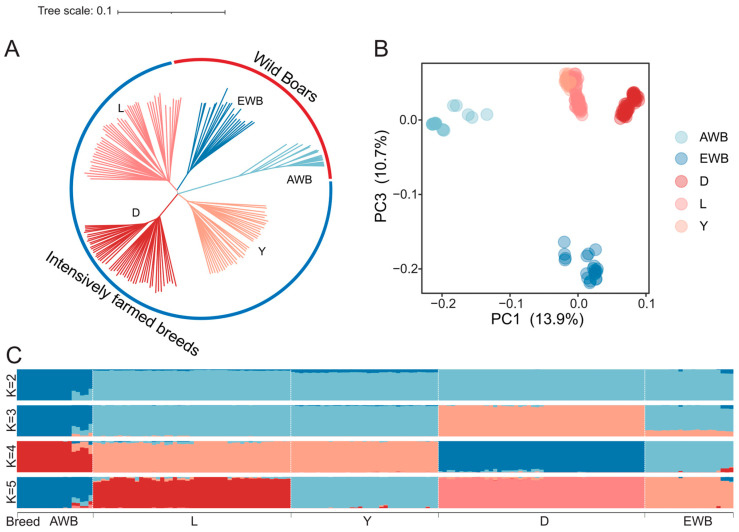
Population genetic structure. (**A**): Principal component analysis (PCA) plot. (**B**): Phylogenetic tree. (**C**): Admixture structure. Abbreviations: AWB = Asian wild boar; D = Duroc; EWB = European wild boar; L = Landrace; Y = Yorkshire.

To investigate the population genetic structure, we conducted PCA, NJ-tree, and admixture analyses. PCA and NJ-tree revealed that intensively farmed breeds (IFBs) tend to be a group separated from EWB and AWB (Figure 2A,B). Admixture analysis is a method used to study the genetic ancestry of different populations by identifying segments of DNA that can be attributed to different ancestral populations. When K = 2, the admixture results revealed that the IFB and EWB groups come together into one cluster, indicating a shared ancestry (Figure 2C). The early differentiation of Duroc pigs from European wild boars at K = 3 suggests a longer history of genetic isolation and potentially unique evolutionary pressures (Figure 2C). The geographical origin of each breed, with Yorkshire and Landrace pigs from the UK and Denmark, respectively, and Duroc pigs from the United States, may have influenced the genetic divergence observed (Figure 2C and Table S1). The distinct genetic clustering of the IFBs from that of European wild boars underscores the impact of domestication and geographical separation on genetic diversity. Finally, to further validate the accuracy of the clustering outcomes, we computed the kinship matrix (Figure S1). Notably, we observed a closer genetic relationship between IFB and EWB, aligning consistently with the findings of the preceding three analyses.

### 3.2. Candidate Genes Under Selection

**Figure 3:**
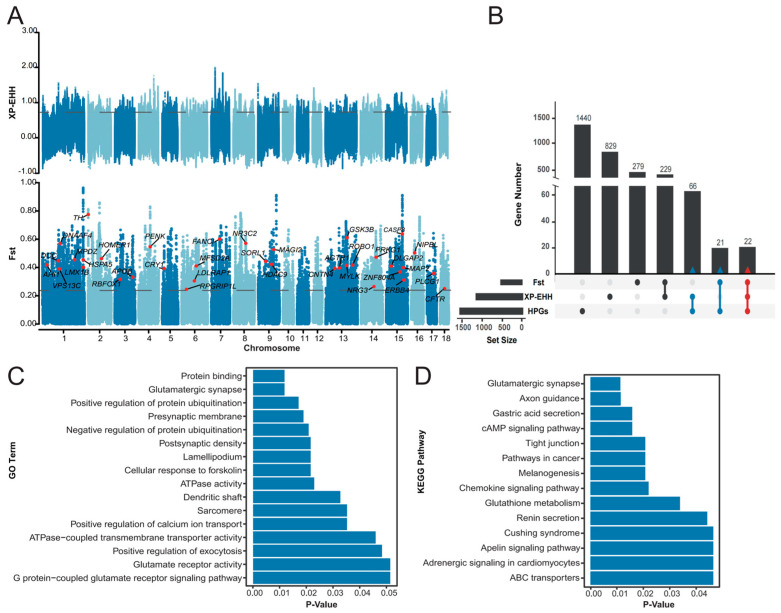
PMH candidate genes. (**A**): Manhattan plots of Fst and XP-EHH, in which the labeled genes were some candidate genes for mental health. (**B**): Venn diagram of the overlapping genes -between the positive selection genes identified through the Fst and XP-EHH methods and the reference genes associated with human mental health. (**C**): Enriched GO terms of PMH candidate genes. (**D**): Enriched KEGG pathways of PMH candidate genes.

In our Fst and XP-EHH analyses, we set a rigorous threshold for outliers within the top 1% of the Fst and XP-EHH values [51] as these parameters are commonly employed in the field and have yielded promising results. Utilizing this criterion, we detected a total of 551 and 1146 candidate genes under selection, respectively (Figure 3A). By combining the gene sets identified through both the Fst test and XP-EHH analysis, we compiled a comprehensive list of 1446 genes that are considered to be under positive selection (Figure 3B).

### 3.3. PMH Candidate Genes

Ultimately, the convergence of genes under positive selection with pig–human homologous genes annotated with human mental disorders led to the identification of a refined set of 109 PMH candidate genes (Figure 3C and Table S3). These genes are now considered prime candidates for further investigation into pig mental health. Fifty-two of these genes overlapped with a set of 254 PMH candidate genes discovered in our other study (unpublished) (Table S3).

### 3.4. KEGG Pathways and GO Biological Processes Enriched with PMH Candidate Genes

To validate the accuracy of the mental health candidate genes obtained through MeHA, we performed a series of functional validations on the candidate genes. A functional enrichment analysis of 109 PMH candidate genes via the KOBAS web platform revealed that PMH candidate genes were over-represented in 30 KEGG pathways (FDR < 0.05) and with GO terms (FDR < 0.05) (Figure 3D and Table S4). These enriched pathways and terms are highly relevant to mental disorders; neuronal protection and development; and processes associated with learning, memory, and behavior.

### 3.5. Disease Enrichment Analysis of PMH Candidate Genes and Eliminated Genes

Upon conducting a disease enrichment analysis on the PMH candidate genes, we identified a total of 56 disease terms (FDR < 0.01), with 8 related to mental health (Table S5). Analyzing the eliminated genes revealed 46 disease terms (FDR < 0.05), with only 4 associated with mental health (Table S6).

### 3.6. Clustered Expression of PMH Candidate Genes in Different Pig Brain Tissues

**Figure 4:**
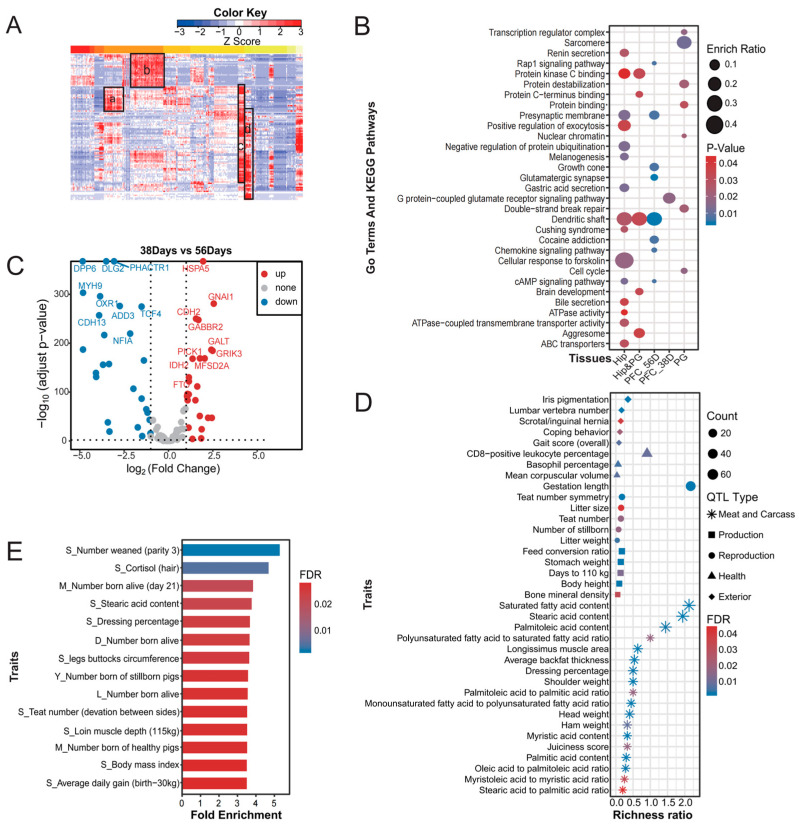
Functional characteristics of PMH candidate genes. (**A**): Tissue-specific gene expression pattern. a: Gene clusters highly expressed in the frontal cortex at 56 days of age. b: Gene clusters highly expressed in the frontal cortex at 38 days of age. c: Gene clusters highly expressed in the hippocampus. d: Gene clusters highly expressed in the pineal gland. (**B**): Enriched GO terms and KEGG pathways of highly expressed gene clusters (see details in Figure S1). (**C**): PMH candidate genes significantly differentially expressed in the cerebral cortex between day 38 and 56. (**D**): QTLs overlapping with PMH candidate genes. (**E**): Enriched traits of PMH candidate genes.

On the basis of their expression profiles in different pig brain tissues from Pig-GTEx data, PMH candidate genes were clustered into four groups (Figure 4A and Figure S2 and Table S7). For each group, we utilized the KOBAS web server to identify KEGG pathways and GO terms (Figure 4B).

The first group, which included 18 genes (a-cluster), was actively expressed in the frontal cortex at 56 days of age. An enrichment analysis identified eight significantly enriched pathways (FDR < 0.01). These pathways include the glutamatergic synapse pathway, which is involved in the regulation of learning, memory, and cognition. Other pathways, such as the chemokine pathway, are associated with neural development. The second group of 24 genes (b-cluster) was expressed almost exclusively in the frontal cortex at 38 days of age. These genes were enriched in the G protein-coupled glutamate receptor signaling pathway (FDR < 0.05), which has been linked to anxiety, stress, sleep disorders, and depression. The third group, consisting of 79 genes, presented high expression in the hypothalamus. These genes were enriched in twenty-two significant pathways (FDR < 0.05), including pathways such as gastric acid secretion, which can affect memory, cognition, and circadian rhythms, as well as pathways that help maintain homeostasis [52]. The fourth group of 37 genes was actively expressed in the pineal gland. Seven significant pathways (FDR < 0.05) related to the regulation of circadian rhythms, memory, and cognition were enriched. Finally, 37 genes shared between the third and fourth groups were enriched in five significant pathways (FDR < 0.05), which play crucial roles in neurodevelopmental protection and are closely associated with circadian rhythm and memory, such as the protein C-terminus-binding pathway.

Additionally, we conducted a comparative analysis of the frontal cortex expression data using 56-day-old pigs as the standard reference and 38-day-old pigs as the experimental group subjected to weaning stimulation. This choice was made due to both age groups being representative of weaned piglets, with the added advantage that pigs at 38 days old have recently undergone the stress of weaning and separation from sows [51]. We observed that 28 genes, including *NFIA*, *DLG2*, *CDH13*, and *TCF4*, were highly expressed on day 38, whereas 25 genes, such as *FTO*, *IDH2*, *HSPA5*, and *MFSD2A*, exhibited high expression on day 56 (Figure 4C).

To gain insight into the interplay among these genes within the cellular context, we subsequently constructed separate protein‒protein interaction (PPI) networks for the four gene clusters. Our aim was to identify key regulatory genes that play crucial roles within these gene clusters. Consequently, we identified the hub gene with the strongest association with the other genes in each gene cluster. *SHANK2* was determined to be the hub gene for the a-cluster, *TIAM1* for the b-cluster, and *GSK3B* for the c-cluster; notably, *GSK3B* was also the hub gene for the d-cluster (Figure S3).

### 3.7. Correlations Between the PMH Candidate Genes and Economic Traits

To investigate the potential impact of PMH candidate genes on pig economic traits, we employed QTL mapping and GWAS signal enrichment analysis. Initially, we executed the QTL mapping analysis on the PMH candidate genes, revealing a substantial number of QTL regions linked to key traits such as the meat and carcass quality, exterior appearance, reproductive success, production efficiency, and health (Figure 4D and Table S8). Notably, many of these QTL regions are associated with meat quality and carcass quality, indicating a shared genetic basis for PMH and production traits. We subsequently conducted a GWAS signal enrichment analysis on the PMH candidate genes, identifying the top 5% of enriched traits, which included the piglet weaning survival count, cortisol levels, litter size, stearic acid content, and dressing percentage (Figure 4E and Table S9). These findings point to a significant potential impact of these genes on traditional reproductive practices, meat quality, and overall health metrics in pig production. In summary, our findings suggest a substantial association between the genes implicated in mental health and a spectrum of production traits, offering valuable insights for genetic improvement programs in pig farming.

## 4. Discussion

An intensive farming system, characterized by a fully enclosed management system with stringent disease prevention and scientific protocols, offers significant advantages. These include enhanced production efficiency, improved economic outcomes, and a reduced environmental impact, making it an optimal approach for stabilizing the pork market. Moreover, new factors, such as high stocking densities, weaning, or population mixing or transfer, can induce stress in pigs [5], potentially leading to behavioral issues such as tail biting, aggression, and postpartum depression. These stresses can also negatively impact productivity metrics such as growth, reproduction, and meat quality [25]. Therefore, understanding the genetic basis of animal mental health traits and applying this knowledge to animal genetic improvement contributes not only to enhancing pig production traits, but also to their overall well-being. However, unlike mental disorders in humans, there is currently no effective way to define and measure mental health traits. Consequently, studies aimed at identifying genes and understanding the biological mechanisms underlying mental health traits have lagged. To address this challenge, we developed a cross-species analysis framework designed to identify potential genetic factors associated with animal mental health traits.

The major concept of the MeHA framework is based primarily on understanding the characteristics of genomic changes from wild animals to intensively farmed animals and using prior knowledge of human mental disorder gene annotations to identify genes related to animal mental health. First, we suppose that intensively farmed animals, such as pig breeds, have evolved through long-term selective breeding to develop traits that make them more resilient to the stresses of the farming environment. Consequently, these breeds may exhibit reduced susceptibility to stress and mental disorders compared with their wild counterparts in similar intensive farming systems. For example, studies have revealed correlations between specific gene expression patterns in the brain and selectable behaviors during pig domestication [53,54]. Genes subject to positive selection have shown significant changes as wild boars have evolved into modern intensively farmed breeds [55]. Building on this foundation, we hypothesize that the domestication process of wild animals into modern intensively farmed breeds has led to developments in brain function, potentially improving memory and cognitive abilities. This evolutionary adaptation might have shaped the genetic foundation of mental health traits. Thus, selection signal analysis can reveal genomic regions under selection to identify mental health candidate genes. Second, by leveraging the human‒animal homology analysis and referencing genes that are well annotated for human mental disorders, we can further refine the identification of mental health candidate genes. This dual-pronged approach, which combines selection signal analysis with cross-species homology, offers a robust way to understand the genetic basis of mental health in animals.

The ability of MeHA to identify mental health candidate genes in pigs and its potential application to other species highlights its importance in advancing animal welfare research. Using the MeHA framework, we identified 109 PMH candidate genes. The discovery of many well-known human–pig homologous genes related to human disorders supports the reliability of our approach. For example, the *SCN2A* and *GSK3B* genes are associated with major depressive disorder and schizophrenia in the Han Chinese population [56,57]. The *FTO* and *ERBB4* genes are involved in depressive-like behaviors and play essential roles in these behaviors induced by various factors [58,59]. Additionally, the *DCC* gene is associated with major depressive disorder, schizophrenia, and bipolar disorders [60].

The enriched pathways involving PMH candidate genes are crucial for synaptic transmission, neural protection, development, memory, and cognition. The associations between these pathways and well-documented neurological processes provide strong support for the credibility of these genes as being related to pig mental health and can offer insights into improving their well-being. For example, the most significantly enriched term was the cellular response to forskolin. Forskolin is known for its neuroprotective effects and potential for Alzheimer’s disease treatment [61]. In transgenic APP/PS1 mice, it improves social behavior and reduces the number of Aβ plaques in the brain [61]. It also enhances synaptic transmission and memory formation [62]. Furthermore, PMH displays heightened expression in pathways associated with the amino acid metabolism, notably the G protein-coupled glutamate receptor pathway. Glutamate, the essential primary excitatory neurotransmitter for memory and learning, regulates neural signals to enhance cognitive processes [63,64,65]. Additionally, the glutamatergic synapse pathway, highly active in both GO terms and KEGG pathways, plays a crucial role in regulating numerous brain functions, including learning, memory, cognition, and development [66]. Enriched pathways like dendritic shafts contribute to the rapid and precise transmission of information in the brain and the maintenance of normal neural function [67]. The incomplete development of dendritic spines has been associated with conditions such as autism and communication disorders [68].

In the disease enrichment results of PMH candidate genes, there are a total of eight terms related to mental health (FDR < 0.01). In the analysis of eliminated genes, only two terms are associated with mental health, including “Neuroticism” and “Nervous system diseases”, both with FDR values significantly exceeding 0.01. Furthermore, the FDR values for these eight prominently expressed terms in the analysis of PMH candidate genes are markedly lower compared to those in the analysis of eliminated genes. This further underscores the enhanced precision in identifying genes related to mental health through MeHA and solidifies the credibility of this approach.

PMH candidate genes are overexpressed in the frontal cortex, hypothalamus, and pineal gland. These regions are known to be associated with human mental disorders. For example, the frontal cortex is integral to emotional regulation and cognitive function [69]. A diminished gray matter volume in the frontal cortex is observed in schizophrenia patients [70]. It is also linked to obsessive‒compulsive disorder, depression, and circadian rhythms, which influence mood and behavior [71,72].

The comparison of the expression of PMH candidate genes in the prefrontal cortex between 38-day-old and 56-day-old pigs offers insights into the neurodevelopmental and stress-related changes occurring in piglets during these periods. At 38 days, piglets have recently been weaned, experiencing psychological stress due to separation from their mothers, leading to potential emotional anxiety, stress, sleep disorders, and even depression. Interestingly, we also observed that 28 genes actively expressed at this stage, including genes such as *NFIA*, *CDH13*, *TCF4*, and *DLG2*, were associated with intellectual disabilities, autism spectrum disorders, irritability, and depression [73,74,75,76,77]. These genes suggest that the stress of weaning may trigger the expression of genes related to emotional and cognitive challenges, supporting the observed behavioral changes, such as anxiety and sleep disturbances. At 56 days, piglets are in an active period for neurodevelopment, memory formation, and cognitive activities and need to recognize and memorize their feeders and living areas. Twenty-five genes have high expression at this stage, including genes such as *PICK1* [78] and *GABBR2* [79], which are thought to be involved in learning and memory, and *GNAI1* [80] and *MFSD2A* [81], which are implicated in neural development. These findings indicate that the genes expressed at 56 days are crucial for piglet neurodevelopment and cognitive activities, aligning with their need to adapt to new environments and learn essential survival skills. The differential expression of PMH candidate genes between day 38 and day 56 in piglets supports the hypothesis that weaning stress on day 38 leads to the activation of genes associated with emotional and cognitive disorders, whereas the genes highly expressed on day 56 are involved in neurodevelopment, learning, and memory. This temporal expression pattern reflects the ability of piglets to adapt from a stress response to active cognitive development, providing valuable insights into their mental health and development.

PPI network analysis also revealed that the hub genes for each gene cluster are associated with specific functions and mental health conditions. For example, *SHANK2*, as the hub gene for a cluster, is a scaffolding protein of the postsynaptic density (PSD) in excitatory neurons that plays a critical role in synaptic formation, maintenance, and function [82]. It is related to emotional regulation, and its dysfunction is implicated in various psychiatric disorders, including autism spectrum disorders, schizophrenia, intellectual disabilities, and bipolar affective disorders [83,84]. Moreover, *TIAM1*, as the hub gene for the b-cluster, promotes the formation and growth of spines and synapses. Aberrations in this gene can result in developmental delay, intellectual disabilities, and epileptic seizures [85]. Additionally, *GSK3B* serves as the hub gene for c-clusters and d-clusters and is involved in numerous physiological processes [86]. It is closely associated with neuroinflammation and several psychiatric disorders, including Alzheimer’s disease, depression, and bipolar affective disorder [87].

Our study provides new evidence that pig mental health is significantly associated with other economic traits under intensive farming systems. The QTL enrichment analysis revealed that PMH candidate genes enriched QTLs such as the meat and carcass quality, exterior appearance, reproductive success, production efficiency, and health, indicating a strong association with these economic traits. Moreover, the GWAS signal enrichment analysis revealed that PMH candidate genes were enriched in reproductive and meat quality traits such as the cortisol levels, litter size, and stearic acid content. These findings are consistent with those of previous studies indicating that stress and mental disorders arising from intensive farming environments can adversely affect these traits, leading to reductions in preweaning survival rates and litter sizes [88]. Additionally, cortisol, a key indicator of health, has been strongly associated with the dysregulation of circadian rhythms and mental health [89]. Specifically, flatter diurnal cortisol slopes have been linked to imbalances in circadian rhythm regulation and the development of mental disorders [90]. In terms of meat quality, the stearic acid content and slaughter rate are important production traits. Compared with wild boars, intensely farmed breeds have been selectively bred for increased leanness and body weight [91], resulting in significant differences in meat quality.

Overall, the MeHA is designed for identifying mental health candidate genes in animals under intensive farming systems. Here, we applied the framework to pigs as a case study. The functional characterization of our identified PMH candidate genes in terms of KEGG pathways, GO biological processes, gene expression patterns, and associations with economic traits revealed their strong relevance to mental health, confirming the feasibility of our method. Its principles and methodologies can be extended and adapted for studying the mental health of other animals under intensive farming systems. This highlights the potential for our approach to contribute to a broader understanding of animal welfare across different species and farming practices.

## 5. Conclusions

In this study, we developed a computational framework called the MeHA to explore the evolution of mental health in animals. We identified 109 genes related to pig mental health, which are linked to memory, cognition, and neural development. These genes also affect traits such as meat quality. Our findings highlight the importance of genetic research in understanding animal behavior, improving farming practices, and providing insights into mental health in both pigs and humans.

## Data Availability

The original contributions presented in the study are included in the article/Supplementary Material, further inquiries can be directed to the corresponding author.

## Supplementary Materials

The following supporting information can be downloaded at: https://www.mdpi.com/article/10.3390/biology13100843/s1. Table S1. Summary information of 170 samples. Table S2. Gene list of human mental disorders. Table S3. PMH candidate genes. Table S4. GO terms and KEGG pathways enriched for PMH candidate genes. Table S5. The disease enrichment results of pig mental health candidate genes. Table S6. The disease enrichment results of eliminated genes. Table S7. The four gene clusters were highly expressed in different tissues. Table S8. Summary information of QTLs overlapping with PMH candidate genes. Table S9. Enriched traits for PMH candidate genes. Figure S1. Heatmap of the kinship matrix. Figure S2. Heatmap of tissue-specific gene expression. The *x*-axis represents tissues, and the *y*-axis represents genes. Figure S3. Protein‒protein interaction networks of four highly expressed gene clusters. A: PPI networks of the frontal cortex at 56 days. B: PPI networks of the frontal cortex at 38 days. C: PPI networks of the hypothalamus. D: PPI networks of the pineal gland.

## Abbreviations

AWB	Asian wild boars
D	Duroc
EWB	European wild boar
GO	Gene Ontology
GWAS	Genome-wide association study
IBS	Identical-by-state
IFBs	Intensively farmed breeds
KEGG	Kyoto Encyclopedia of Genes and Genomes
L	Landrace
LD	Linkage disequilibrium
MAF	Minor allele frequency
PCA	Principal component analysis
PMH	Pig mental health
PPI	Protein‒protein interaction
PSD	Postsynaptic density
QTL	Quantitative Trait Locus
WGS	Whole-genome sequencing
Y	Yorkshire
