# Supplementary material for: MeHA: A Computational Framework in Revealing the Genetic Basis of Animal Mental Health Traits Under an Intensive Farming System—A Case Study in Pigs
Source: Biology (Basel). 2024 Oct 21;13(10):843. doi: 10.3390/biology13100843 (PMC11504952; doi:10.3390/biology13100843)
Supplement: Supplementary file 1 [file biology-13-00843-s001.zip › biology-3208403-supplementary.pdf]

Figure S1. Heatmap of the kinship matrix.

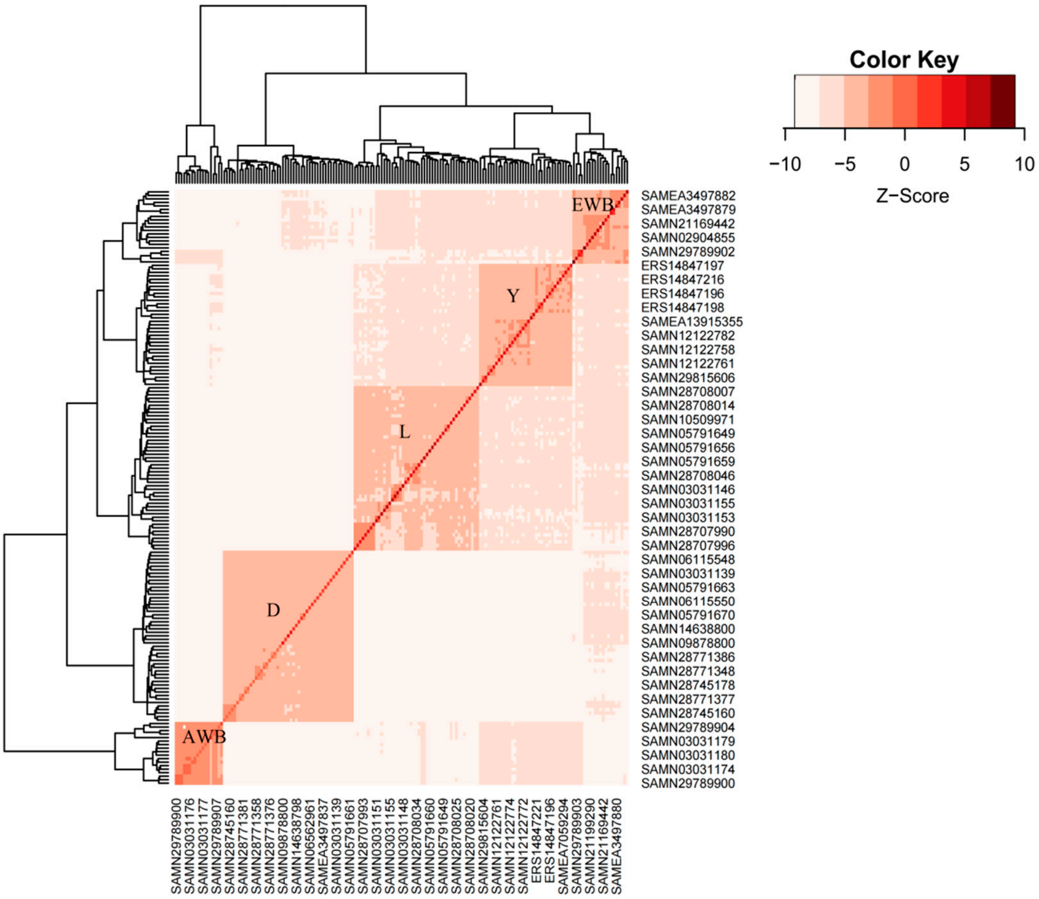

Figure S2. Heatmap of tissue-specific gene expression. The x-axis represents tissues, and the y-axis represents genes.

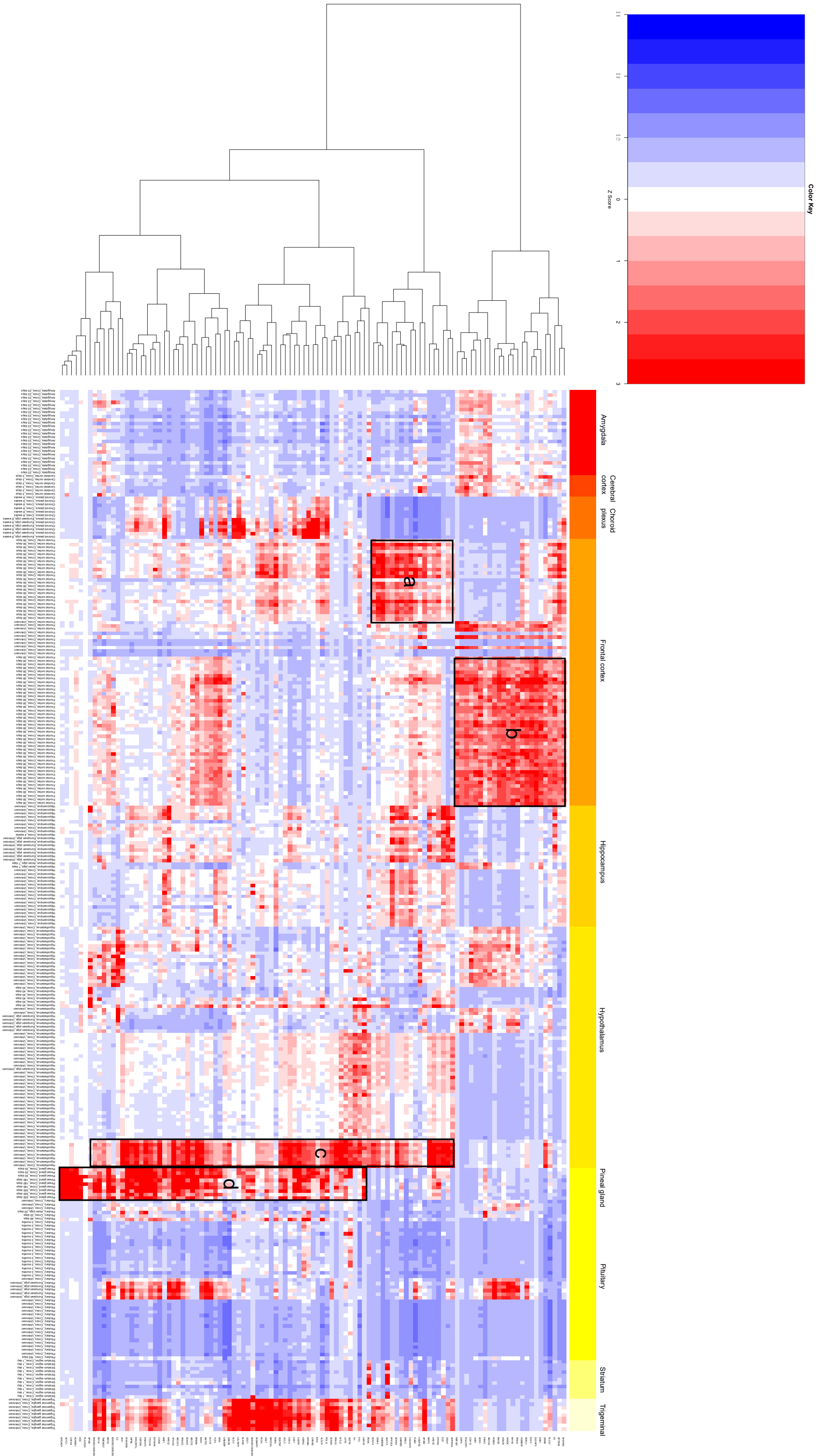

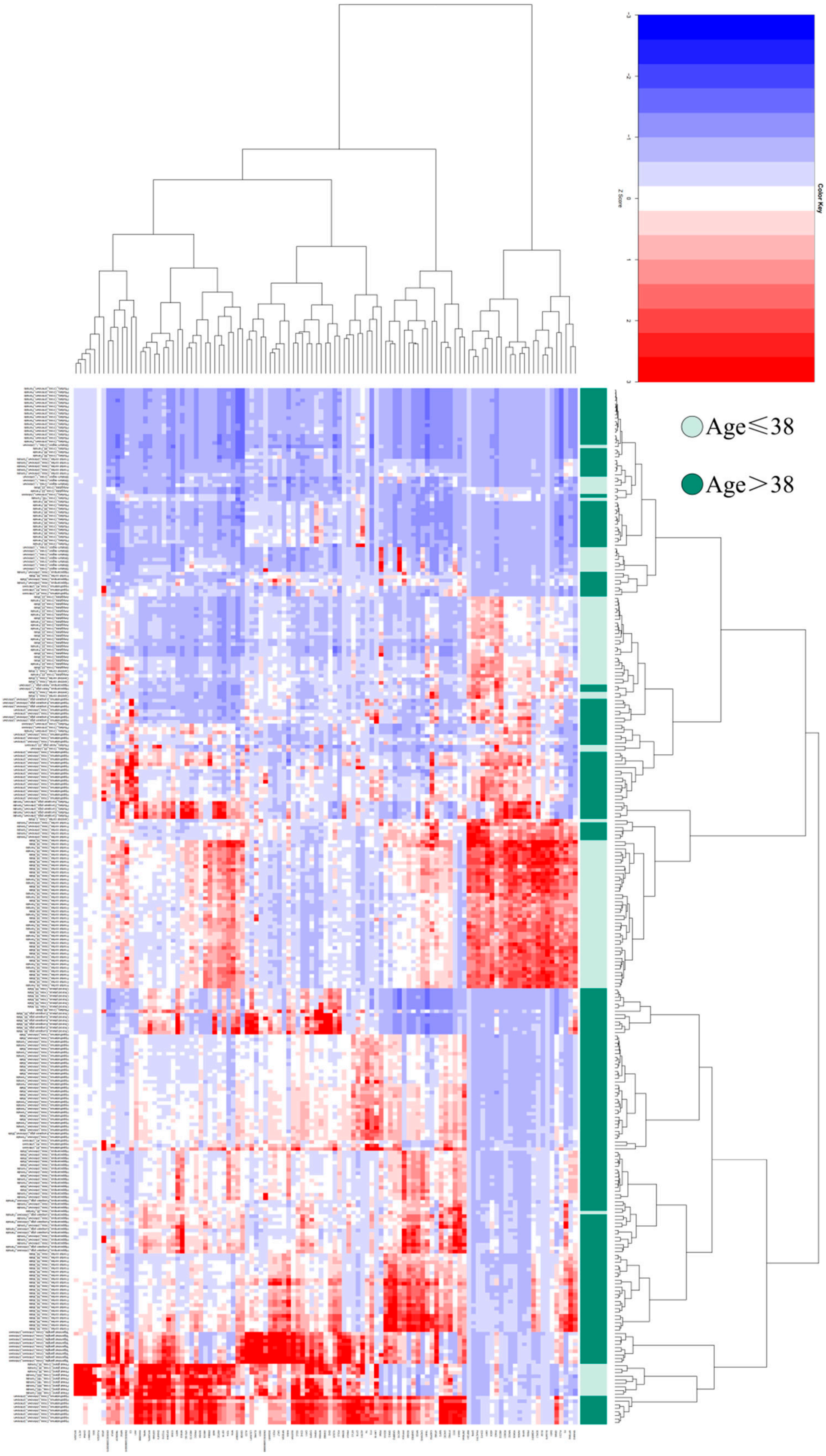
Age≤38
Age>38

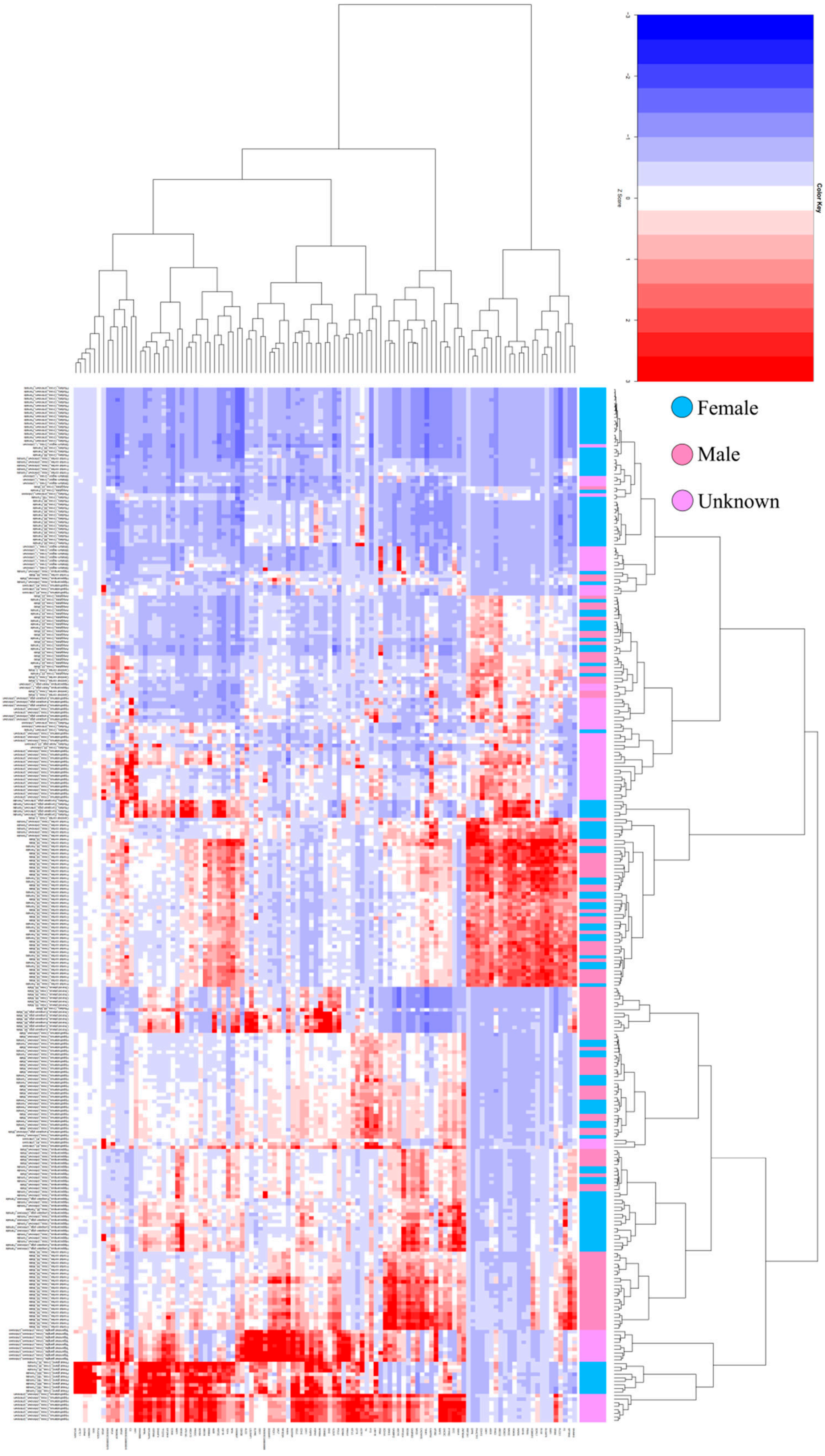
Female
Male
Unknown

Figure S3. Protein-protein interaction networks of four highly expressed gene clusters.

A

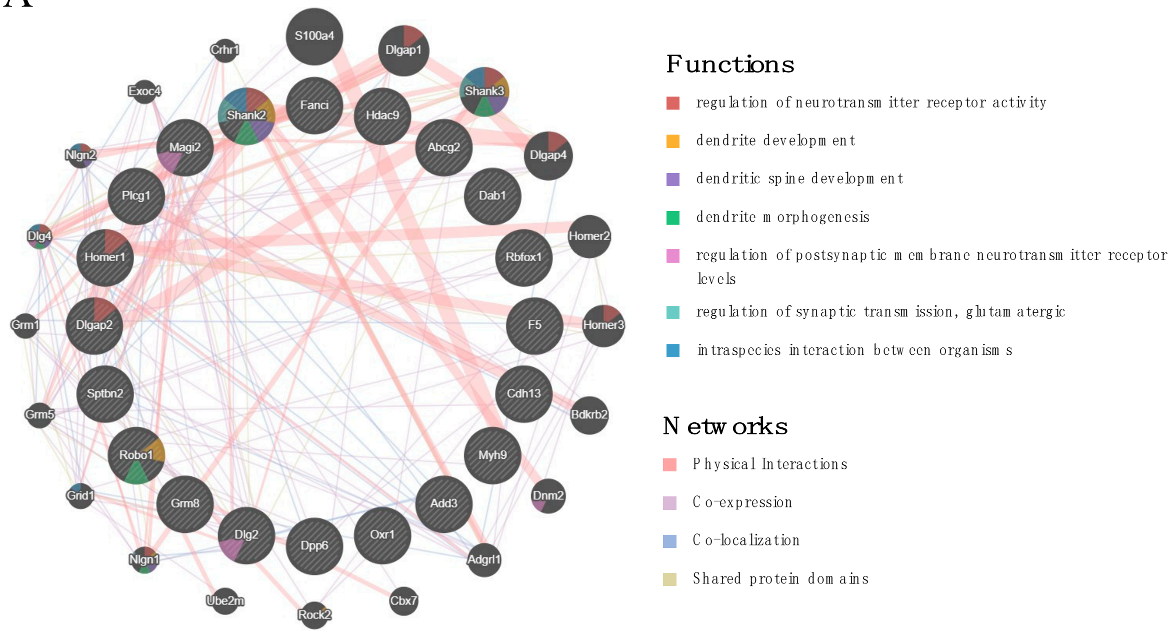

B

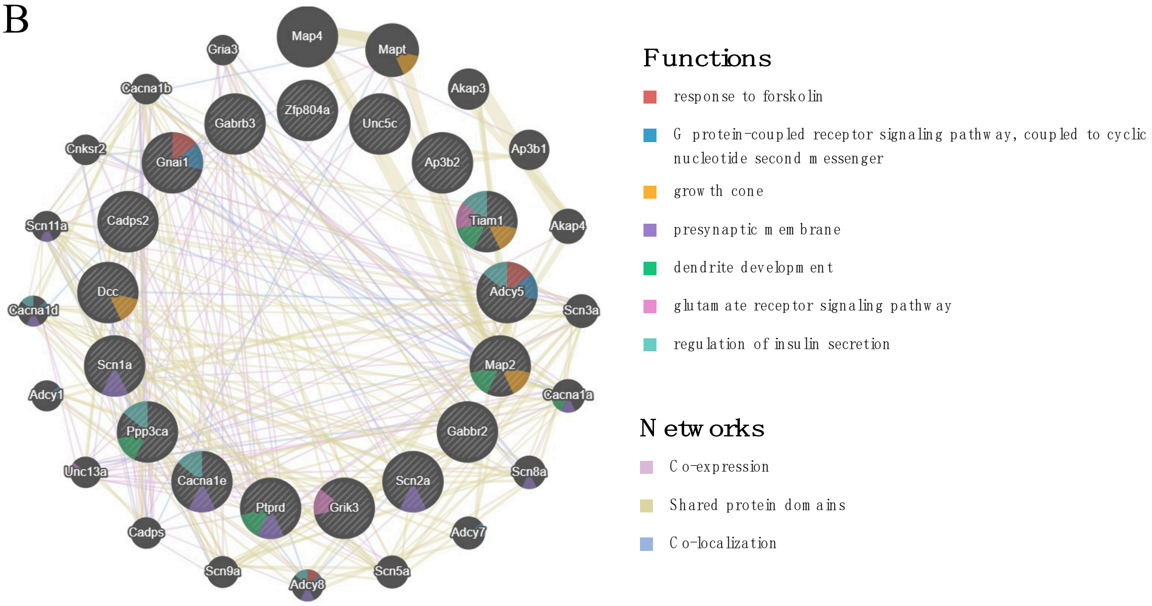

C

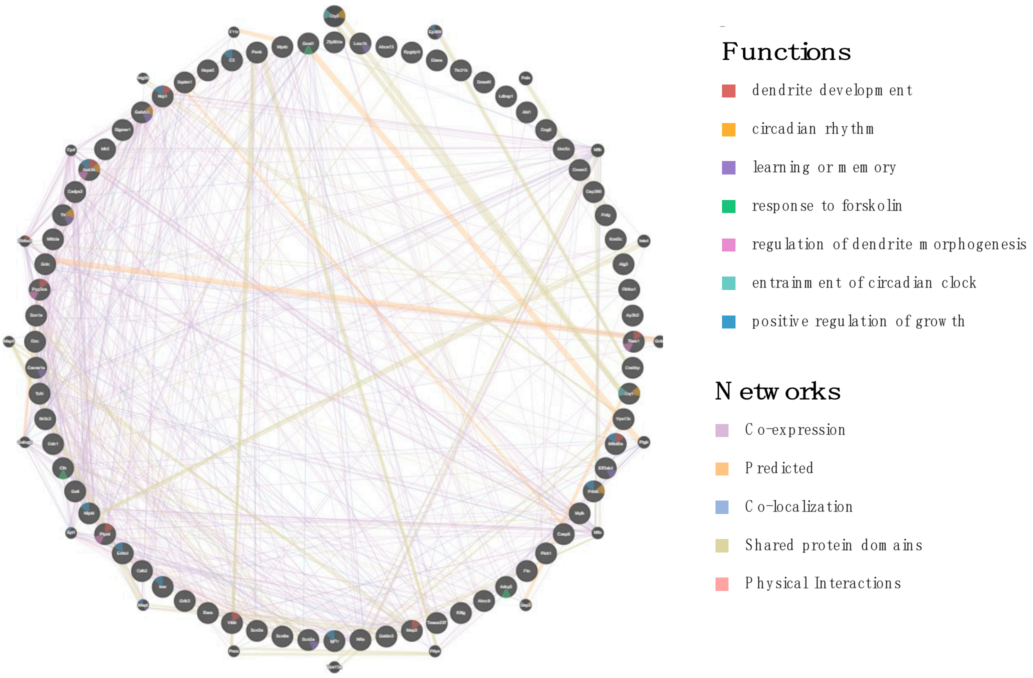
Functions
dendrite development
circadian rhythm
learning or memory
response to forskolin
regulation of dendrite morphogenesis
entrainment of circadian clock
positive regulation of growth
Networks
Co-expression
Predicted
Co-localization
Shared protein domains
Physical Interactions

D

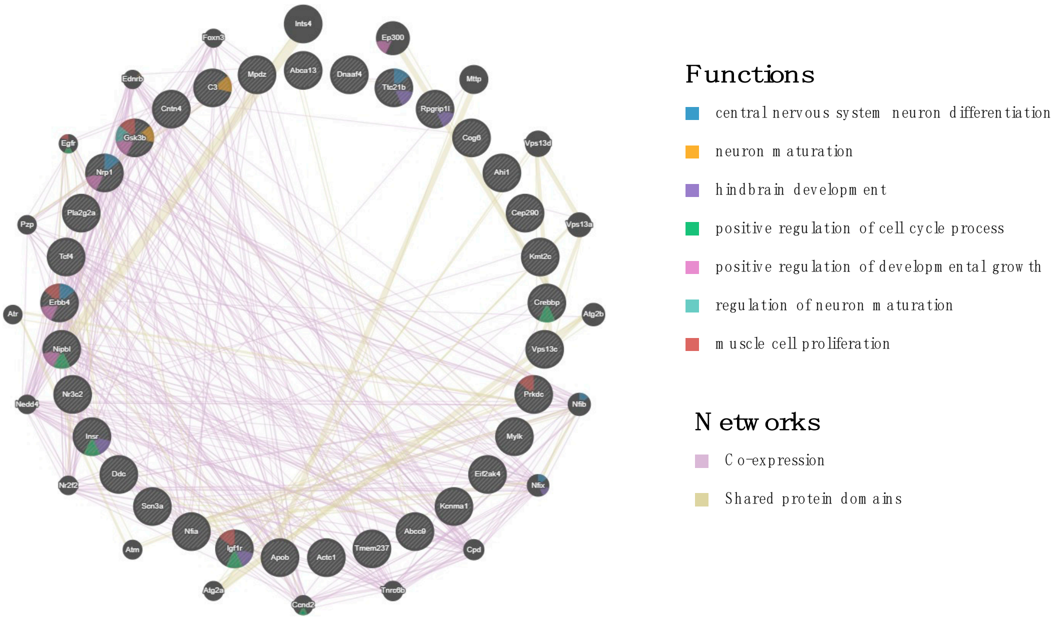
Ints4
Foxn3
Ep300
Mpdz
Abca13
Dnaaf4
Mttp
Ednrb
C3
Ttc21b
Cntn4
Rpgrip1l
Gsk3b
Cog6
Vps13d
Egfr
Ahi1
Nrp1
Pla2g2a
Cep290
Vps13a
Pzp
Tcf4
Kmt2c
Erbb4
Crebbp
Atr
Atg2b
Nipbl
Vps13c
Nedd4
Nr3c2
Prkdc
Nfib
Insr
Mylk
Ddc
Eif2ak4
Nfix
Nr2f2
Scn3a
Kcnma1
Nfia
Abcc9
Cpd
Atm
Igf1r
Apob
Actc1
Tmem237
Atg2a
Tnrc6b
Ccnd2
Functions
central nervous system neuron differentiation
neuron maturation
hindbrain development
positive regulation of cell cycle process
positive regulation of developmental growth
regulation of neuron maturation
muscle cell proliferation
Networks
Co-expression
Shared protein domains
